# Round the Hospitals

**Published:** 1916-09-23

**Authors:** 


### ROUND THE HOSPITALS.

A meeting to further the College of Nursing movement is announced to be held at Mildmay Hut, Salisbury Road, Plymouth, on Friday, September 22, after we go to press. Colonel Webber, C.O. 4th Southern General Hospital, is to be in the chair, and Miss Rundle and Miss Gibson are to speak. These meetings, which are being held up and down the country, are most useful in making known the objects and work of the College. Last week the Hon. Arthur Stanley spoke at two in Scotland (at Glasgow on September 14 and Edinburgh on September 15), and the previous week at one in Brighton. Each was interesting, well attended, and thoroughly representative. At the Edinburgh meeting Mr. Stanley said that the keynote of the College of Nursing movement was that the organisation of the nursing profession should come from within. The amount of such organisation was enormous—questions of curriculum, of examinations, and of the standard of training had to be settled, and surely nurses themselves, and those particularly interested in the profession, must be best fitted to settle them. State recognition and registration of nurses would protect the members of the profession and the public. At present any woman who likes can call herself a trained nurse, and dress as though she were one. The Bill will provide for the State recognition of uniform or of some kind of badge. Anyone wearing this without the right to do so will be penalised. Expert advice has been sought and given free of charge in the preparation of the Bill for the State Registration of Nurses, which has been moulded in the form most likely to pass through Parliament. Mr. Stanley said he hoped that very shortly either he or Major Chapple would present the new measure to the House of Commons. Mr. Stanley in his Brighton speech emphasised the desirability of going to Parliament with 10,000 names on the Register, and reminded his hearers that Registration Offices had been opened at the College of Nursing, 6 Vere Street, London, W.

---

At all meetings stress was laid on the point that the charge for nurses registering as members of the College would be fixed at a guinea, and there would be no further payment to make. It was difficult to enumerate the specific advantages which would accrue to the nurses, but there was a paramount advantage to be gained by belonging to a large and powerful body. Cases were on record where nurses had received unjust treatment in going out to private cases. This sort of treatment would stop if behind the nurses there were a strong body like the College. It was hoped to have a large building devoted to the College in a central part of London, a site for this being at present available. The cost was £50,000. Of course this could not come out of the nurses' guineas, but Mr. Stanley believes that at this time, when the nurses have shown themselves so ready to sacrifice everything in caring for the sick and wounded, there are many ready to help with the building of a suitable College of Nursing. He had only approached four people, but they had given him £200, £500, £1,000, and 1,000 guineas respectively!

---

Various questions raised at the Edinburgh meeting had mainly to do with the relation of Scotland to the College. It was pointed out that the names of Scottish nurses would not only appear on the Central Register, but on the Sectional Scottish Register, and that thus the nurses would elect their own Scottish Board. Dr. Leslie Mackenzie, medical member of the Local Government Board, showed that there already existed in Scotland a Register for Poor-Law and fever-trained nurses, and Dr. Robertson pointed out that there was also a Register of mental nurses for the whole of the United Kingdom. What would be the relation of the College to these Registers? Mr. Stanley said that this matter would be fully threshed out by the Council of the College, and stated that the fact that the Local Government Board recognised certain forms of training practically assured their recognition by the College.

---

The Poor-Law Infirmary Matrons' Association now includes seventy-four matrons and superintendent nurses. The matrons of practically all the Metropolitan infirmaries belong to it, and most of those in the country. Miss Todd, at present on foreign service, matron of St. John's Infirmary, is honorary secretary; Miss Alsop, matron of the Kensington Infirmary, is the honorary assistant secretary; and the honorary treasurer is Miss Cockerell, matron of St. Marylebone Infirmary. It is of the utmost importance to Poor-Law nursing and nurses that this Association should include all qualified matrons and superintendents who are trained and certificated nurses. It is matter for congratulation that Miss Barton, the president, should be holding "at homes" at the Chelsea Infirmary fortnightly. The series included an "at home" on the 15th instant, and there will be others on September 30 and October 14 and 28 respectively, which any members of the Poor-Law Infirmary Matrons' Association are cordially invited to attend. These meetings enable the members to keep in touch with each other, a plan which has important bearings seeing the number of interesting and urgent questions affecting trained nurses which must come up for settlement during the autumn and winter months. The training in the best of the Poor-Law infirmaries has produced excellent results and given a high standard of reputation to certificated nurses trained in these infirmaries, who are usually both popular and highly efficient, in private nursing especially.

---

We regret that owing to pressure on our space the reply to our correspondent's letter published in last week's issue, on Mental Nurses in Military Hospitals, is unavoidably held over.

For Matrons' and Sisters' Appointments, see p. 590. For "The College of Nursing: A Glasgow View," see p. 587.

